# THE ROAD TO ACCEPTANCE OF DRIVER RETIREMENT FOR PATIENTS WITH DEMENTIA: PHYSICIANS’ AND PATIENTS’ PERSPECTIVES

**DOI:** 10.1093/geroni/igz038.430

**Published:** 2019-11-08

**Authors:** Theresa L Scott, Jacki Liddle, Nancy A Pachana, Elizabeth Beattie, Geoffrey Mitchell

**Affiliations:** 1 The University of Queensland, Queensland, Australia; 2 Queensland University of Technology, Queensland, Australia

## Abstract

People living with Alzheimer’s disease and related dementias (ADRD) must eventually stop driving. While some will voluntarily retire, many others will continue to drive until a crisis. In Australia, like many other countries, general physicians/practitioners (“GPs”) play a key role in monitoring driving safety and driver retirement with their patients with ADRD. Advising patients about driving cessation is one of the most challenging aspects of clinical dementia care, complicated by limited time in consultations, lack of patient awareness and insight, and objective screening and assessment measures. We examined how to support best practice in relation to management of driving cessation with patients with ADRD through focus groups with 29 GPs and contrasted their perspectives with those of 11 retired drivers with ADRD. Focus groups and interviews were transcribed and thematically analysed. Themes discovered highlighted the importance of providing education about the effects of dementia on safe driving and incorporating regular assessment of driving safety into the care continuum. Key strategies that GPs successfully employed included acknowledging loss and encouraging continued community engagement, providing referral pathways, and deferring to other GPs within the practice in challenging circumstances. In conclusion, there is demand for an overhaul of the current system of management and a need to establish nationally aligned, standardized and evidence-based guidelines, in particular relating to assessment of safe driving. In the meantime, we can learn from these GPs who have implemented particular strategies that mitigate some of the challenges and complex driving related issues that present in primary care.

